# Measures to control protracted large Lassa fever outbreak in Nigeria, 1 January to 28 April 2019

**DOI:** 10.2807/1560-7917.ES.2019.24.20.1900272

**Published:** 2019-05-16

**Authors:** Chioma C. Dan-Nwafor, Yuki Furuse, Elsie A Ilori, Oladipupo Ipadeola, Kachikwulu O Akabike, Anthony Ahumibe, Winifred Ukponu, Lawal Bakare, Tochi J Okwor, Gbenga Joseph, Nwando G Mba, Adejoke Akano, Adebola T Olayinka, Ihekerenma Okoli, Rita A Okea, Favour Makava, Nkem Ugbogulu, Saliu Oladele, Geoffrey Namara, Esther N Muwanguzi, Dhamari Naidoo, Samuel K Mutbam, Ifeanyi Okudo, Solomon F Woldetsadik, Clement LP Lasuba, Chikwe Ihekweazu

**Affiliations:** 1 Nigeria Centre for Disease Control, Abuja, Nigeria; 2 These authors contributed equally to this work; 3 World Health Organization, Abuja, Nigeria; 4 Institute for Frontier Life and Medical Sciences, Kyoto University, Kyoto, Japan; 5 United States Centers for Disease Control and Prevention, Abuja, Nigeria; 6 Georgetown University, Washington DC, United States of America; 7 Federal Ministry of Agriculture and Rural Development, Abuja, Nigeria; 8 Federal Ministry of Environment, Abuja, Nigeria; 9 Maryland Global Initiatives Corporation, Abuja, Nigeria; 10 Pro Health International, Abuja, Nigeria

**Keywords:** Lassa fever, Nigeria, Disease outbreaks, Epidemiology, Public health

## Abstract

Lassa fever cases have increased in Nigeria since 2016 with the highest number, 633 cases, reported in 2018. From 1 January to 28 April 2019, 554 laboratory-confirmed cases including 124 deaths were reported in 21 states in Nigeria. A public health emergency was declared on 22 January by the Nigeria Centre for Disease Control. We describe the various outbreak responses that have been implemented, including establishment of emergency thresholds and guidelines for case management.

Lassa fever is endemic in Nigeria. From 1 January 2019 (week 1) to 28 April 2019 (week 17), 554 laboratory-confirmed Lassa fever cases including 124 deaths were reported from 21 of 37 states/territory in Nigeria. On 22 January, Nigerian public health authorities declared the outbreak and emergency. Here, we describe the current situation and implemented response to the outbreak.

## Current outbreak and surveillance of Lassa fever

From 2016 to 2018, 177, 312 and 633 confirmed Lassa fever cases were reported, respectively, in Nigeria. While the outbreak recorded the largest ever number of cases in 2018, the number of confirmed cases between 1 January and 28 April 2019 has surpassed that of the corresponding period in 2018; the number of suspected cases, including laboratory-negative cases, also surpassed that for the same period (Table and Figure). Blood samples were collected for all suspected cases and tested for Lassa fever using reverse transcription PCR. Positivity rate among suspected cases in this outbreak was 24%, similar to that of previous years (Table). The increase in confirmed cases in 2019 could result, at least partially, from improved disease surveillance systems.

**Table:** Summary of statistics of Lassa fever outbreak in Nigeria, 1 January 2017–28 April 2019 (n = 1,499 confirmed cases)

Week	Total suspected cases	Positive rate (%)	Confirmed cases										
			Total	Deaths	Case-fatality rate (%)	Healthcare workers	Ebonyi state	Edo state	Ondo state	Bauchi state	Plateau state	Taraba state	Other states
**2017**													
1–17	462	32	149	53	36	NA	5	56	23	6	8	23	28
1–52	1,030	30	312	78	25	NA	5	116	77	13	21	24	56
**2018**													
1–17	1,891	22	420	106	25	37	63	176	99	10	9	19	44
1–52	3,506	18	633	171	27	45	72	279	159	24	14	23	62
**2019**													
**1–17**	**2,323**	**24**	**554**	**124**	**22**	**18**	**44**	**197**	**155**	**42**	**35**	**38**	**43**

NA: not available.

**Figure:**
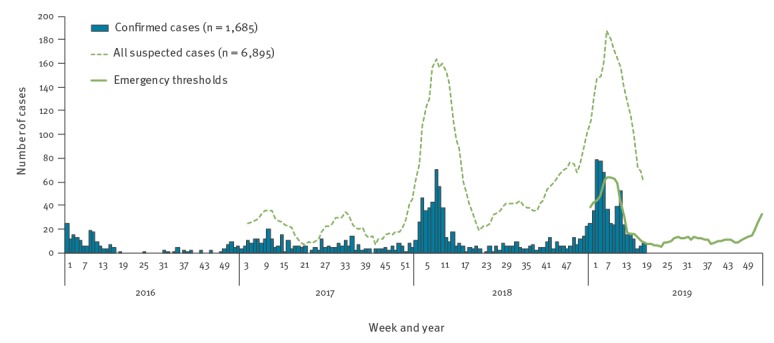
Number of Lassa fever cases in Nigeria by calendar week, 1 January 2016–28 April 2019 (n = 1,685 confirmed cases)

Weekly numbers of confirmed cases from 2016–19, all suspected cases including confirmed and laboratory-negative cases from 2017–19 and emergency thresholds for 2019 were plotted. Data of all suspected cases in 2016 were not available.

The main reservoir for of Lassa fever virus is rodents and humans can be infected by contact with infected animals or their excretions, although human-to-human transmission is possible and sporadically reported [1]. Analysis of viral genomic sequence of samples collected in 2019 revealed that the main source of infection is spill over from rodents [2].

The Nigeria Centre for Disease Control (NCDC) was established in 2011 and has strengthened the surveillance system for infectious diseases, including Lassa fever [3]. On 22 January 2019, the NCDC declared the Lassa fever outbreak an emergency in response to the high case numbers. Surveillance training was provided for public health officers at both national and state levels. Sensitisation to increase suspicion of the disease was conducted for healthcare workers through workshops and for the general public through media such as radio, television, poster and social network services. Strengthening of laboratory capacity and sample transportation system has also facilitated disease detection and reporting through establishment of the Lassa fever laboratory network.

## Clinical management and new guidelines

The fatality rate among confirmed cases was 22% (124/554) in 2019. National and zonal workshops for clinical management of Lassa fever patients were convened as a part of outbreak response. Furthermore, national guidelines to treat Lassa fever patients were reviewed and updated in November 2018, to include infection prevention and control (IPC) measures, guidance on clinical treatment and care of complications after Lassa fever infection e.g. septic shock and kidney injury [4]. These recommendations arose following findings that such complications are important risk factors for fatal outcome among Lassa fever patients [5]. Unfortunately, there has been no substantial change in the case-fatality rate over the past 3 years (Table). Familiarising healthcare workers with the new guidelines and helping them to better detect and treat Lassa virus earlier [6], should help improve the prognosis and reduce the mortality of the disease.

Healthcare workers are at risk of infection from Lassa fever patients when adherence to IPC is poor [1]. In 2019, there have been 18 healthcare workers infected with Lassa fever (as at 28 April). In the guidelines, a full set of personal protective equipment is recommended for healthcare workers who provide direct patient care to suspected or confirmed Lassa fever patients. While necessary supplies have been distributed to Lassa fever treatment centres in 2019 (due to improved communication and logistics system in the country), the resource-limited setting in Lassa fever endemic areas is still challenging in terms of feasibility and sustainability.

## Determining thresholds for emergency detection

As Lassa fever is endemic in Nigeria and cases are reported every year, it becomes more important to monitor changes in the trend of infection or an abnormally high number of cases. With this in mind, emergency designation criteria thresholds were developed using statistical criteria that account for expected epidemiology of disease patterns in Nigeria (Figure). Briefly, the threshold was developed as the mean plus two standard deviations of the weekly number of confirmed cases for the period of 2 weeks before and 2 weeks after the week of interest over the past 3 years (i.e. data from 2016 to 2018 for the development of thresholds for 2019). Although the methodology did not use environmental variables e.g. rainfall and population of rodents, the thresholds captured seasonal fluctuation by referring to historical data. By monitoring exceedances of current case numbers against these thresholds, abnormal trends can be identified to determine the emergency phase of outbreak.

## Situation and potential of domestic and international spread of the disease

The majority of confirmed cases (396/554, 71%) in 2019 were reported from three states with standard Lassa fever treatment centres (Ebonyi, Edo and Ondo); Lassa fever treatment centres are also situated in other states. It is unknown whether the prevalence of Lassa fever was considerably high in those three states, whether there was heightened suspicion of Lassa fever enforcing local surveillance activity, or both. Interestingly, other states e.g. Bauchi, Plateau and Taraba, showed the highest historical number of cases in each state in 2019 (Table). This also makes the situation in 2019 unusual and could suggest emergency measures are required.

Lassa fever was included as a notifiable disease under the International Health Regulations due to its epidemic prone nature and potential to cause economic and social disruption [7]. There was a probable exportation of Lassa fever cases from Nigeria to Benin in 2018. Three confirmed cases were reported in Benin who had migrated from Nigeria to Benin just before the detection of the disease (data not shown). Although exportation of Lassa fever cases has not been reported so far in 2019, confirmed cases were reported in Nigerian states adjacent to Benin, Cameroon and Niger.

Nigeria is the host of the West African regional coordinating centre of the Africa Centres for Disease Control and Prevention (Africa CDC, Addis Ababa, Ethiopia) recently established in 2017 [8], this means that there is a strong collaborative relationship with the NCDC. Although there was no deployment need from the Africa CDC during the Lassa fever outbreak in 2019, there had been deployments to support Lassa fever response in Nigeria, in 2017. In 2019, the regional body supported through virtual meetings, to understand the situation in Nigeria and assess the level of risk to other African countries.

The main outbound travel destinations from Nigeria to Europe include France and the United Kingdom (UK) [9], and the number of travellers from the UK to Nigeria are ca 150,000 per year (second only to Niger). Given the overall large number of international flights from Nigeria, exportation of the disease outside of the African continent including European countries is possible in theory. Still, the World Health Organization does not recommend any travel or trade restriction to Nigeria based on the currently available information [10].

Healthcare workers outside Nigeria should be aware of the situation in case they receive patients with compatible clinical symptoms and history of a stay in areas in Nigeria where there is transmission of the disease.

## Conclusion

Here, we described the situation of the Lassa fever outbreak in Nigeria as at 28 April 2019. In addition to the improved surveillance system, there could be climatic or ecological conditions influencing the large waves in 2018 and 2019. Utilisation of data of environmental factors will possibly allow us to predict outbreaks of the disease in the future. We must remain vigilant and keep improving disease surveillance and clinical management for future outbreaks. Healthcare workers must have a high index of suspicion of the disease and adhere to IPC measures while providing care for all patients. Familiarising health care workers with the new guidelines mentioned above is also important to minimise the risk of nosocomial transmission of Lassa fever.
